# Supplementary material for: The Genetic Determinants of Extreme UV Radiation and Desiccation Tolerance in a Bacterium Recovered from the Stratosphere
Source: Microorganisms. 2025 Mar 27;13(4):756. doi: 10.3390/microorganisms13040756 (PMC12029717; doi:10.3390/microorganisms13040756)
Supplement: Supplementary file 1 [file microorganisms-13-00756-s001.zip › Ellington_SuppMat_v3.pdf]

Supplementary Materials for:

The Genetic Determinants of Extreme UV Radiation and Desiccation Tolerance in a Bacterium Recovered from the Stratosphere

Adam J. Ellington [1,2], Tyler J. Schult [1], Christopher R. Reisch [1,3] and Brent C. Christner [1,*]

[1] Department of Microbiology and Cell Science, Institute of Food and Agricultural Science, University of Florida, Gainesville, FL 32611, USA
[2] Meso Scale Diagnostics, LLC, Rockville, MD 20850, USA
[3] Genomatica, San Diego, CA 92121, USA
* Correspondence: xner@ufl.edu

This section includes:

Supplementary Methods
References for Supplementary Materials
Supplementary Tables S1-S7
Supplementary Figures S1-S6

Supplemental Materials and Methods

Directed Evolution and Mutation Analysis

The survival of strain DSM 20129 to UVCR exposure was determined as described above. Triplicate colonies surviving the highest UVCR dose were selected, grown separately in liquid media, exposed to UVCR (as described in the section "UVCR Survival Assays"), and the process was repeated a total of 12 times (Fig. 4A). The populations from each cycle of exposure were archived by freezing aliquots of the cultures in 25% glycerol and storing at -70°C.

Genome sequences were obtained using the Illumina NextSeq 2000 sequencing platform (Microbial Genome Sequencing Center, Pittsburgh, PA) with 2×150 bp reads for select strains throughout the directed evolution process where an increase in UVCR tolerance was observed (i.e., after rounds 1, 3, 6, 7, and 9). Sequencing reads were quality filtered and adaptors trimmed using the Trim Galore script [58], followed by mapping to the reference genome and mutation identification using the default parameters of the Breseq mutation analysis pipeline set to consensus mode [74]. The ancestral parent strain was sequenced and used as a control to correct the reference genome before comparison with the evolved strains. Gene Ontology (GO) functional annotations for proteins incurring mutations throughout the directed evolution process were obtained using Blast2GO [65].

Structural Prediction and Analysis of Curtobacterium Photolyases

Sequence alignments were performed using the Clustal Omega multiple sequence alignment tool [156] for the photolyases found in strains L6-1 and DSM 20129 with known bacterial CPD and (6-4) photolyases. Structural predictions for these photolyases were also made using AlphaFold2 [157] and structural alignments were created with the jFATCAT (rigid) algorithm [158, 159] in the Protein Data Bank (PDB) pairwise structure alignment tool.

Cloning and Expression of Curtobacterium Photolyases

To assess the performance of the *Curtobacterium* photolyases independent from additional variables present in each of the individual host contexts, each was cloned into an expression vector for expression in *E. coli* BL21. The plasmids pDLxR6 and pCLxR4 were used for expression and contain antibiotic selection markers for spectinomycin and carbenicillin, respectively. These vectors place expression of the target gene under control of the N-(3-oxohexanoyl) homoserine lactone (OC6)-inducible P_{luxB}/LuxR promoter/repressor system and were shown to be comparable to T7 expression systems while enabling tighter control over leaky expression [160]. The primer sequences used for PCR amplification and cloning are found in Table S6. Briefly, primers with overhangs encoding regions of homology between the vector and insert amplicons were used to PCR amplify the vectors and photolyase genes from the chromosomes of strains L6-1 and DSM 20129 using Q5 High-Fidelity 2x Master Mix (New England Biolabs, NEB) as follows: L6-1 *phr1* (KM842_RS01465; primers 2942 + 2943), L6-1 *phr2* (KM842_RS02105; primers 2940 + 2941), DSM 20129 *phr* (K0028_16065; primers 2938 + 2939), pDLxR6 vector (primers 2872 + 2040), pCLxR4 vector (primers 2872 + 2953). PCR amplification was followed by DpnI digestion to remove template DNA, agarose gel electrophoresis to verify amplicon size, and column-based DNA cleanup. DNA fragments were then assembled using the 2x NEBuilder HiFi DNA Assembly Master Mix (NEB) with the following modifications: 1 μL Master Mix + 0.5 μL each DNA fragment, incubated at 50°C for 15 min, yielding pDLxPhr02105, pDLxPhr16065, and pCLxPhr01465. Chemically competent *E. coli* NEB5a cells were used for cloning. The *E. coli phrB* gene, encoding the native CPD photolyase, was also cloned into the pDLx backbone to serve as a control, yielding pDLxEcPhrB. All plasmids were verified by Sanger sequencing and transformed into competent

E. coli BL21 cells via electroporation. 5 mL cultures of BL21 cells harboring the photolyase expression vectors were grown to mid log-phase (OD_{600} ~0.4-0.5) in LB + appropriate antibiotics at 37°C, 250 rpm. Photolyase expression was then induced by adding OC6 (1 μM), followed by an additional two hours of growth. Photolyase activity was determined as described above.

References for Supplemental Information*

*Listed according to numbering in main text

14. Bryan NC, Christner BC, Guzik TG, Granger DJ, Stewart MF. 2019. Abundance and survival of microbial aerosols in the troposphere and stratosphere. ISME Journal 13:2789–2799.
44. Bryan NC, Stewart M, Granger D, Guzik TG, Christner BC. 2014. A method for sampling microbial aerosols using high altitude balloons. J Microbiol Methods 107:161–168.
58. Krueger F. 2021. TrimGalore: A wrapper around Cutadapt and FastQC to consistently apply adapter and quality trimming to FastQ files, with extra functionality for RRBS data. GitHub.
65. Götz S, García-Gómez JM, Terol J, Williams TD, Nagaraj SH, Nueda MJ, Robles M, Talón M, Dopazo J, Conesa A. 2008. High-throughput functional annotation and data mining with the Blast2GO suite. Nucleic Acids Res 36:3420–3435.
74. Deatherage DE, Barrick JE. 2014. Identification of mutations in laboratory-evolved microbes from next-generation sequencing data using breseq. Methods in Molecular Biology 1151:165–188.
156. Sievers F, Wilm A, Dineen D, Gibson TJ, Karplus K, Li W, Lopez R, McWilliam H, Remmert M, Söding J, Thompson JD, Higgins DG. 2011. Fast, scalable generation of high-quality protein multiple sequence alignments using Clustal Omega. Mol Syst Biol 7:539.
157. Jumper J, Evans R, Pritzel A, Green T, Figurnov M, Ronneberger O, Tunyasuvunakool K, Bates R, Žídek A, Potapenko A, et. al. 2021. Highly accurate protein structure prediction with AlphaFold. Nature 596:583–589.
158. Ye Y, Godzik A. 2003. Flexible structure alignment by chaining aligned fragment pairs allowing twists. Bioinformatics 19:ii246–ii255.
159. Li Z, Jaroszewski L, Iyer M, Sedova M, Godzik A. 2020. FATCAT 2.0: towards a better understanding of the structural diversity of proteins. Nucleic Acids Res 48:W60–W64.
160. Schuster, L.A.; Reisch, C.R. Plasmids for Controlled and Tunable High-Level Expression in *E. coli*. *Appl. Environ. Microbiol*. 2022, *88*, e00939-22. https://doi.org/10.1128/aem.00939-22.

Table S1: Strains used in this study

Strain	Reference/Source
Curtobacterium aetherium L6-1	[14,44]
Curtobacterium flaccumfaciens str. DSM 20129	DSMZ: DSM20129
Mycetocola reblochoni str. LMG 22367	NRRL: B-24476
Plantibacter flavus str. DSM 14012T	DSMZ: DSM14012
Escherichia coli str. K-12 substr. MG1655	
Escherichia coli str. BL21	
Deinococcus radiodurans R1	

Table S2: UVC survival curves and the predicted dose required to reach the LD90 (J m^{-2}).

**Strain**	**k (± SEM, m^2 J^{-1})**	**R^2 (± SEM)**	**LD90 (± SEM, J m^{-2})**	**LD90$_{Desiccated}$* (J m^{-2})**
Curtobacterium sp. L6-1	-0.0078 (± 0.0041)	0.95 (± 0.02)	469.84 (± 166.45)	1200
Curtobacterium flaccumfaciens DSM 20129	-0.0246 (± 0.0035)	1.00 (± 0.00)	97.97 (± 15.66)	n.d.
Mycetocola reblochoni	-0.0684 (± 0.0068)	1.00 (± 0.00)	34.30 (± 3.14)	n.d.
Plantibacter flavus	-0.0280 (± 0.0017)	0.91 (± 0.08)	82.72 (± 4.74)	n.d.

*Values from Bryan et al. 2019. *ISME J.*
n.d. = not determined

Table S3: DNA repair genes present and absent in *Curtobacterium sp.* L6-1 and *Curtobacterium flaccumfaciens* strain DSM 20129.

Repair System	Gene	Function	NCBI Accession (L6-1)	NCBI Accession (DSM 20129)	Identity to L6-1 (%)
**Base Excision Repair (BER)**					
	alkA	DNA-3-methyladenine glycosylase II	WP_216259360.1[#]	QYI96388.1[#]	81.5
	ligA	NAD-dependent DNA ligase	WP_216257571.1	QYI98502.1	89.2
	mutM	Formamidopyrimidine-DNA glycosylase	WP_216261011.1[+]	QYI97427.1[+]	74.4
	mug	G/U mismatch-specific DNA glycosylase	Not present	Not present	N/A
	mutY	Adenine DNA glycosylase	WP_216262075.1	QYI98925.1	90.1
	mutT	8-oxo-dGTP diphosphatase	WP_216259654.1<br>WP_216259011.1<br>WP_216262173.1	QYI96591.1<br>QYI96134.1<br>QYI98116.1	83.8<br>69.7<br>74.5
	nei	DNA glycosylase/AP lyase (Endonuclease VIII)	WP_216259379.1<br>WP_216257488.1<br>WP_216257855.1<br>WP_216261011.1[+]	QYI96398.1<br>QYI98429.1<br>Not present<br>QYI97427.1[+]	87.6<br>94.2<br>N/A<br>74.4
	nfo	Endonuclease IV	Not present	Not present	N/A
	nth	DNA glycosylase and AP lyase (Endonuclease III)	Not present	Not present	N/A
	polA	DNA polymerase I	WP_216261341.1	QYI97641.1	92.5
	tag	3-methyladenine DNA glycosylase I	WP_216260468.1	QYI97126.1	86.5
	yxlJ (Bs)	3-methyladenine DNA glycosylase	WP_216259816.1	QYI96708.1	75.3
	ung	Uracil DNA glycosylase	WP_216261189.1	QYI97516.1	84.6
	xthA/exoA (Bs)	Exonuclease III	WP_216258313.1<br>WP_216260746.1	QYI98874.1<br>QYI97265.1	90.7<br>89.6
**Nucleotide Excision Repair (NER)**					
	mfd	Transcription-repair coupling factor	WP_216257769.1	QYI98636.1	90.6
	uvrA	ATPase and DNA damage recognition protein of excinuclease UvrABC	WP_216262103.1<br>WP_216261795.1	QYI97651.1<br>QYI97714.1	94.3<br>40.2
	uvrB	Excinuclease ABC subunit B	WP_216261345.1	QYI97649.1	94.7

Repair System	Gene	Function	NCBI Accession (L6-1)	NCBI Accession (DSM 20129)	Identity to L6-1 (%)
	uvrC	Excinuclease ABC subunit C	WP_253206254.1	QYI97652.1	79.6
	uvrD/pcrA (Bs)	DNA helicase II	WP_216259555.1<br>WP_216257614.1<br>WP_216261784.1	Not present<br>QYI98523.1<br>Not present	N/A<br>86.1<br>N/A
	cho	Excinuclease cho	Not present	Not present	N/A
	uvsE (Bs)	UV DNA damage endonuclease	Not present	Not present	N/A
**Mismatch Repair (MMR)**					
	dam	DNA adenine methylase	Not present	Not present	N/A
	mutH	DNA mismatch repair protein MutH	Not present	Not present	N/A
	mutL	DNA mismatch repair protein MutL	Not present	Not present	N/A
	mutS	DNA mismatch repair protein MutS	Not present	Not present	N/A
	nucS≠ *(Mt)*	Mismatch-specific endonuclease NucS	WP_216258989.1	QYI96128.1	92.2
**Homology-directed Repair/Homologous Recombination (HDR/HR)**					
RecBCD Pathway	*recA*	Recombinase recA	WP_216261887.1	QYI98316.1	96.6
	recB	Exonuclease V (RecBCD complex), beta subunit	Not present	Not present	N/A
	recC	Exonuclease V (RecBCD complex), gamma chain	Not present	Not present	N/A
	recD	Exonuclease V (RecBCD complex), alpha chain	Not present	Not present	N/A
RecFOR Pathway	*recF*	DNA replication/repair protein RecF	WP_216259097.1	QYI96198.1	89.4
	recO	DNA repair protein RecO	WP_216261628.1	QYI98032.1	91.7
	recR	Recombination mediator RecR	WP_017888680.1	QYI97228.1	100
	recJ	Single-stranded DNA-specific exonuclease	Not present	Not present	N/A
	recN	DNA repair protein RecN	WP_216259826.1	QYI96712.1	89.9
	recQ	DNA helicase RecQ	WP_216258070.1<br>WP_216259567.1<br>WP_216262198.1	QYI98758.1<br>QYI96526.1<br>QYI98297.1	90.7<br>75.7<br>91.7
Branch Migration	*ruvA*	Holliday junction branch migration protein RuvA	WP_216261438.1	QYI97746.1	80.1
	ruvB	Holliday junction branch migration DNA helicase RuvB	WP_216261437.1	QYI97745.1	92.3

Repair System	Gene	Function	NCBI Accession (L6-1)	NCBI Accession (DSM 20129)	Identity to L6-1 (%)
Resolvases	*ruvC*	Crossover junction endodeoxyribonuclease RuvC	WP_216261439.1	QYI97747.1	78.1
	recU (Bs)	Holliday junction resolvase RecU	Not present	Not present	N/A
	ruvX (Mt)	Holliday junction resolvase RuvX	WP_216262082.1	QYI98926.1	91.2
	recG	ATP-dependent DNA helicase RecG	WP_216262035.1	QYI97422.1	90.2
Other	*radA*	DNA repair protein RadA	WP_216258833.1	QYI96040.1	90.5
	radD	Putative DNA repair helicase RadD	WP_216259721.1	QYI96630.1	90.5
	rmuC	DNA recombination protein RmuC	WP_216257711.1	QYI98595.1	87.1
	recX	Regulatory protein RecX	WP_216261882.1	QYI98315.1	72.6
	addA (Bs)	ATP-dependent helicase/nuclease subunit A	Not present	Not present	N/A
	addB (Bs)	ATP-dependent helicase/nuclease subunit B	Not present	Not present	N/A
	adnA (Mt)	End resection helicase-nuclease subunit A	WP_216257618.1	QYI98527.1	82.8
	adnB (Mt)	End resection helicase-nuclease subunit B	WP_216262265.1	QYI98526.1	83.1
**Nonhomologous End Joining (NHEJ)**					
	ku (Bs, Mt)	DNA DSB-binding protein Ku	WP_216258571.1	QYI95908.1	88.2
	ligC (Mt)	ATP-dependent DNA ligase LigC	WP_216261741.1	QYI98144.1	85.8
	ligD (Bs, Mt)	ATP-dependent DNA ligase LigD	WP_216258565.1<br>WP_216261744.1	QYI95906.1<br>QYI98147.1	78.4<br>84.9
**Direct DNA Damage Reversal**					
	ada/adaA	transcriptional activator Ada	WP_216259360.1[#]	QYI96388.1[#]	81.5
	ogt/adaB	O6-methylguanine-DNA methyltransferase	WP_216259362.1	QYI96389.1	77.7
	alkB	Alpha-ketoglutarate-dependent dioxygenase AlkB	Not present	Not present	N/A
	phr	DNA photolyase	WP_216260506.1	QYI97148.1	78.4

Repair System	Gene	Function	NCBI Accession (L6-1)	NCBI Accession (DSM 20129)	Identity to L6-1 (%)
			WP_216261959.1*	Not present	N/A
	splB (Bs)	Spore photoproduct lyase	WP_216260219.1	QYI96960.1	89.6
**SOS Response and Error Prone Replication**					
	dinB	DNA polymerase IV	WP_216261394.1	QYI97696.1	91.3
	polB	DNA polymerase II	Not present	Not present	N/A
	umuC	Error prone DNA polymerase V subunit C	Not present	Not present	N/A
	umuD	Error prone DNA polymerase V subunit D	Not present	Not present	N/A
	dnaE2 (Mt)	Error-prone DNA polymerase	WP_216257368.1	QYI98368.1	86.0
	dinF	DNA-damage-inducible SOS response protein	WP_216262424.1	QYI99065.1	87.1
	dinG	ATP-dependent DNA helicase	Not present	Not present	N/A
	dinI	DNA damage-inducible protein I	Not present	Not present	N/A
	lexA	Transcriptional repressor LexA	WP_216261861.1	QYI98310.1	94.4
	xseA	Exodeoxyribonuclease VII	Not present	QYI98609.1	N/A
	sbcB	Exodeoxyribonuclease I	Not present	Not present	N/A
	sbcC	Exonuclease SbcCD, subunit C	WP_216258073.1	QYI98762.1	58.3
	sbcD	Exonuclease SbcCD, subunit D	WP_216258075.1	QYI98763.1	76.8
	ssb	Single-stranded DNA-binding protein	WP_216259125.1<br>WP_216257507.1	QYI96216.1<br>QYI98440.1	87.6<br>77.6

(Bs) = alternate repair protein in *B. subtilis*

(Mt) = alternate repair protein in *M. tuberculosis*

#Bifunctional transcriptional activator/DNA-3-methyladenine glycosylase with homology to both AlkA and AdaA

≠Non-canonical mismatch repair enzyme in Actinobacteria

+Bifunctional DNA-formamidopyrimidine glycosylase/AP lyase with homology to both MutM and Nei

*Not homologous to *E. coli* protein, but belongs to the same family

Table S4: ROS detoxification genes present and absent in *Curtobacterium sp.* L6-1 and *Curtobacterium flaccumfaciens* strain DSM 20129.

Gene	Function	NCBI Accession (L6-1)	NCBI Accession (DSM 20129)	Identity to L6-1 (%)
**ROS Scavengers**				
katA (Bs)	Catalase	WP_253206366.1	Not present	N/A
katE	Catalase	Not present	Not present	N/A
katG	Catalase	Not present	Not present	N/A
katX (Bs)	Catalase	Not present	QYI97794.1	N/A
sodA	Superoxide dismutase [Mn]	WP_216260500.1	QYI97144.1	98.0
sodB	Superoxide dismutase [Fe]	Not present	Not present	N/A
sodC	Superoxide dismutase [Cu/Zn]	Not present	Not present	N/A
ahpA (Bs)	Biofilm-specific peroxidase AhpA	Not present	Not present	N/A
ahpC	Alkyl hydroperoxide reductase subunit C	Not present	Not present	N/A
ahpD (Mt)	Alkyl hydroperoxide reductase AhpD	Not present		
ahpE (Mt)	Peroxiredoxin AhpE	WP_216261603.1	QYI98005.1	90.7
ahpF	Alkyl hydroperoxide reductase subunit F	Not present	Not present	N/A
ohrA (Bs)	Organic hydroperoxide resistance protein A	WP_216259992.1	QYI96823.1	87.9
ohrB (Bs)	Organic hydroperoxide resistance protein B	WP_216258174.1	QYI98818.1	88.7
osmC	Peroxiredoxin OsmC	WP_216258661.1	QYI95943.1	91.8
**Transcriptional Regulators**				
oxyR	Hydrogen-peroxide inducible genes activator	WP_216258516.1	QYI95875.1	82.9
soxR	Redox-sensitive transcriptional activator	WP_216261496.1<br>Not present	QYI99079.1<br>QYI95963.1	83.2<br>N/A
soxS	Transcriptional activator of the superoxide response regulon	Not present	Not present	N/A
fur	Iron uptake regulation	Not present	Not present	N/A
zur	Zinc uptake regulation	WP_216258491.1	QYI95859.1	83.3
perR (Bs)	Peroxide operon regulator	WP_216258151.1	Not present	N/A

Gene	Function	NCBI Accession (L6-1)	NCBI Accession (DSM 20129)	Identity to L6-1 (%)
ohrR (Bs)	Organic hydroperoxide resistance transcriptional regulator	WP_216260161.1<br>WP_216259994.1	QYI96935.1<br>QYI96824.1	83.7<br>87.3
fnr	Fumarate and nitrate reduction regulatory protein	Not present	Not present	N/A
sigH (Mt)	ECF RNA polymerase sigma factor SigH	WP_216262048.1	QYI98917.1	85.8
**DNA Protection from Oxidation**				
dps	DNA protection during starvation protein	WP_216258537.1<br>Not present	QYI95891.1<br>QYI97778.1	89.3<br>N/A
**Disulfide Reduction**				
gshB	Glutathione synthase	Not present	Not present	N/A
gshR	Glutathione reductase	WP_216261555.1<br>WP_216261228.1	QYI97896.1<br>QYI97533.1	94.8<br>86.9
gstA	Glutathione S-transferase	Not present	Not present	N/A
btuE	Thioredoxin/glutathione peroxidase	WP_216259497.1	QYI96476.1	87.0
grxA	Glutaredoxin 1	Not present	Not present	N/A
grxB	Glutaredoxin 2	Not present	Not present	N/A
grxC	Glutaredoxin 3	Not present	Not present	N/A
grxD	Glutaredoxin 4	Not present	Not present	N/A
nrdH	Glutaredoxin-like protein NrdH	WP_058769310.1<br>WP_216258763.1	QYI96735.1<br>Not present	98.7<br>N/A
trxA	Thioredoxin	WP_110902894.1<br>WP_216261086.1	QYI96211.1<br>QYI97464.1	94.4<br>81.7
trxB	Thioredoxin reductase	WP_216259121.1<br>Not present<br>WP_216262026.1	QYI96212.1<br>QYI97086.1<br>QYI97393.1	90.3<br>N/A<br>74.1
trxC	Thioredoxin 2	Not present	Not present	N/A
tpx	Thiol peroxidase	Not present	Not present	N/A
bcpA	Thiol peroxidase BCP type	WP_216260919.1<br>WP_216257664.1	QYI97363.1<br>QYI98554.1	87.8<br>79.3
bcpB (Mt)	Peroxiredoxin	Not present		
resA (Bs)	Thiol-disulfide oxidoreductase ResA	WP_216261984.1	QYI97165.1	72.4
msrA	Methionine sulfoxide reductase MsrA	WP_216258754.1<br>WP_216257508.1	QYI96017.1<br>QYI98442.1	86.0<br>89.7
msrB	Methionine sulfoxide reductase MsrB	WP_216259705.1	QYI96619.1	95.4
**Other**				

Gene	Function	NCBI Accession (L6-1)	NCBI Accession (DSM 20129)	Identity to L6-1 (%)
mntH	Divalent metal cation transporter	WP_216259399.1	QYI96410.1	84.3
pqiA	Paraquat inducible protein A	Not present	Not present	N/A
pqiB	Paraquat inducible protein B	Not present	Not present	N/A
mshA (Mt)	D-inositol 3-phosphate glycosyltransferase	WP_216261746.1<br>WP_216261460.1	QYI98149.1<br>QYI97768.1	87.0<br>79.9
mshB (Mt)	N-acetyl-1-D-myo-inosityl-2-amino-2-deoxy-alpha-D-glucopyranoside deacetylase	WP_216257737.1<br>WP_216257649.1	QYI98619.1<br>QYI98548.1	83.3<br>78.8
mshC (Mt)	L-cysteine:1D-myo-inositol 2-amino-2-deoxy-alpha-D-glucopyranoside ligase	WP_216261401.1	QYI97703.1	84.5
mshD (Mt)	Mycothiol acetyltransferase	WP_216262484.1	QYI96594.1	76.1
mtr (Mt)	Mycothiol reductase	Not present	Not present	N/A
mscR (Mt)	S-nitrosomycothiol reductase	WP_216257794.1	QYI98495.1	78.0

(Bs) = alternate detoxification protein in *B. subtilis*
(Mt) = alternate detoxification protein in *M. tuberculosis*

Table S5: Mutations identified by Breseq in the UV-evolved DSM 20129 strains.

Location	Position	Mutation	Strains w/ Mutation						Annotation	Gene	Description
			WT	1	3	6	7	9			
Chr	7,692	G→C		X	X	X	X	X	D180H (GAC→CAC)	*K0028_00025* →	NAD(P)/FAD-dependent oxidoreductase
Chr	72,765	T→C				X	X	X	F122L (TTC→CTC)	*trmD* →	tRNA (guanosine(37)-N1)-methyltransferase TrmD
Chr	160,708	C→A					X	X	P183T (CCG→ACG)	*K0028_00715* →	uracil-DNA glycosylase
Chr	205,567	G→A					X	X	E84K (GAG→AAG)	*prfA* →	peptide chain release factor 1
Chr	217,440	C→T						X	H457Y (CAC→TAC)	*atpD* →	F0F1 ATP synthase subunit beta
Chr	559,194	C→G				X	X	X	Q15H (CAG→CAC)	*K0028_02565* ←	flagellar hook capping protein
Chr	612,632	C→A						X	A37S (GCC→TCC)	*K0028_02820* ←	RNA polymerase sigma factor
Chr	682,540	C→G				X	X	X	A171P (GCC→CCC)	*K0028_03160* ←	GrpB family protein
Chr	1,015,248	T→C				X	X	X	K13R (AAG→AGG)	*K0028_04710* ←	cold-shock protein
Chr	1,073,811	G→A					X	X	G170S (GGC→AGC)	*K0028_05010* →	carbohydrate-binding protein
Chr	1,173,658	G→T		X	X	X	X	X	R410L (CGG→CTG)	*K0028_05480* →	HAMP domain-containing histidine kinase
Chr	1,448,846	G→C						X	A343G (GCG→GGG)	*K0028_06725* ←	transaldolase family protein
Chr	1,750,735	2 bp→CT				X	X	X	coding (162-163/1803 nt)	*K0028_08150* ←	S8 family serine peptidase
Chr	2,439,407	G→A				X	X	X	D78N (GAC→AAC)	*K0028_11565* →	L-serine ammonia-lyase
Chr	2,583,948	C→A						X	V95F (GTC→TTC)	*coaA* ←	type I pantothenate kinase
Chr	2,778,691	A→G		X	X	X	X	X	L78P (CTG→CCG)	*K0028_13275* ←	phosphotransferase
Chr	3,362,910	A→G						X	W367R (TGG→CGG)	*K0028_16065* ←	DNA photolyase family protein
Chr	3,363,495	G→T						X	P172T (CCC→ACC)	*K0028_16065* ←	DNA photolyase family protein
Plasmid	42,326	T→G						X	V108G (GTG→GGG)	*K0028_17640* →	inorganic diphosphatase
Plasmid	63,755	G→A		X	X	X	X	X	W396* (TGG→TGA)	*metK* →	methionine adenosyltransferase
**Hypothetical Proteins**											
Chr	91,508	T→A				X	X	X	Y487F (TAC→TTC)	*K0028_00425* ←	hypothetical protein
Chr	132,251	C→T						X	R475Q (CGG→CAG)	*K0028_00590* ←	hypothetical protein
Chr	1,444,899	G→A					X	X	A84A (GCC→GCT)	*K0028_06700* ←	hypothetical protein
Chr	1,839,196	T→C			X	X	X	X	K534R (AAG→AGG)	*K0028_08545* ←	hypothetical protein
Chr	2,549,056	+30 bp		X	X	X	X	X	coding (626/1185 nt)	*K0028_12095* →	hypothetical protein
Plasmid	113,554	T→C						X	E177G (GAG→GGG)	*K0028_18015* ←	hypothetical protein
**Intergenic**											
Chr	3,329,295	C→G		X	X	X	X	X	intergenic (+15/-18)	*nadA* → / → *nadB*	quinolinate synthase NadA/L-aspartate oxidase

Location	Position	Mutation	Strains w/ Mutation						Annotation	Gene	Description
			WT	1	3	6	7	9			
Chr	3,372,508	G→A					X	X	intergenic (+23/-87)	*K0028_16115* → / → *K0028_16120*	helix-turn-helix domain-containing protein/AURKAIP1/COX24 domain-containing protein
Chr	143,018	G→A					X	X	intergenic (-144/+13)	*K0028_00620* ← / ← *K0028_00625*	hemolysin family protein/NADH:flavin oxidoreductase/NADH oxidase
Chr	824,516	A→G				X	X	X	intergenic (-710/+64)	*dnaE* ← / ← *K0028_03835*	DNA polymerase III subunit alpha/RluA family pseudouridine synthase
Chr	2,420,587	A→C					X	X	intergenic (-75/-93)	*K0028_11450* ← / → *K0028_11455*	DUF4832 domain-containing protein/ThuA domain-containing protein
**Pseudogene**											
Chr	1,954,411	A→G					X	X	pseudogene (1165/1821 nt)	*K0028_09115* →	glycoside hydrolase family 15 protein
Chr	1,954,653	G→A					X	X	pseudogene (1407/1821 nt)	*K0028_09115* →	glycoside hydrolase family 15 protein
Chr	432,555	$(C)_{5\to4}$		X	X	X	X	X	pseudogene (480/727 nt)	*K0028_01925* →	aquaporin family protein
**Silent Mutations**											
Chr	60,507	C→T					X	X	F15F (TTC→TTT)	*ffh* →	signal recognition particle protein
Chr	473,119	G→A						X	V428V (GTC→GTT)	*K0028_02110* ←	carboxypeptidase regulatory-like domain-containing protein
Chr	1,005,977	G→C			X	X	X	X	L182L (CTC→CTG)	*K0028_04660* ←	ABC transporter ATP-binding protein
Chr	2,058,688	C→T				X	X	X	G143G (GGC→GGT)	*K0028_09655* →	M81 family metallopeptidase
Chr	2,420,772	C→T			X	X	X	X	I31I (ATC→ATT)	*K0028_11455* →	ThuA domain-containing protein
Chr	2,955,576	G→A				X	X	X	T206T (ACC→ACT)	*K0028_14145* ←	Asp23/Gls24 family envelope stress response protein

Table S6: List of primers used in this study.

Primer	Sequence
2040	5′-GCTTTCCAGTCGGGAAACCTGCTGCCACCGCTGA-3′
2872	5′-GGTGATGGTGCATCTAGTATTTCTCCTCTTTAATCTGCGCTCTTCCCAGTTC-3′
2938	5′-TACTAGATGCACCATCACCATCACCACGGCGGTGGCGGTTCGGTGACCGGCGCGATCGT-3′
2939	5′-AGGTTTCCCGACTGGAAAGCTCACGAGCGCCGCATCCGTT-3′
2940	5′-TACTAGATGCACCATCACCATCACCACGGCGGTGGCGGTTCGATGAGCGGCTCCGTCGT-3′
2941	5′-AGGTTTCCCGACTGGAAAGCTCATGCGCGTCGCATATCGG-3′
2942	5′-TACTAGATGCACCATCACCATCACCACGGCGGTGGCGGTTCGATGGTCGTCGTCGAGGC-3′
2943	5′-AGGTTTCCCGACTGGAAAGCGTTGATCGATCCCACGGGGC-3′
2953	5′-GCTTTCCAGTCGGGAAACCTGCTTTCCAGTCGGGA-3′
2965	5′-TACTAGATGCACCATCACCATCACCACGGCGGTGGCGGTTCGATGATTACCCATCTGGTCTG-3′
2966	5′-AGGTTTCCCGACTGGAAAGCTTATCTGGCTCCTTTCCTCG-3′

Table S7: Read counts (in millions) for each condition throughout the preprocessing and mapping pipeline.[1]

Category	Condition						
	Control	UVCR Exposure		Desiccation			
	t = 0	no recovery	20 min recovery	75% H_2O remaining	50% H_2O remaining	25% H_2O remaining	0% H_2O remaining
**Raw Reads**	15.866 ±0.190	17.269 ±1.104	15.614 ±1.642	16.611 ±0.936	16.001 ±0.322	14.571 ±1.628	16.245 ±1.003
**After Adaptor Trimming and Quality Filtering**	14.129 ±0.466	16.451 ±1.131	14.970 ±1.579	16.077 ±1.147	15.523 ±0.515	13.919 ±1.446	16.050 ±1.001
**Unmapped[2]**	0.657 ±0.379	0.618 ±0.306	0.653 ±0.190	0.853 ±0.588	1.226 ±0.858	1.428 ±0.861	1.811± 0.945
**Mapped**	13.472 ±0.638	15.834 ±0.889	14.316 ±1.449	15.224 ±1.143	14.297 ±1.362	12.491 ±2.266	14.239 ±1.945
**Assigned to Features**	12.478 ±0.480	14.515 ±0.912	13.116 ±1.458	14.219 ±1.198	13.587 ±1.360	12.089 ±2.234	13.801 ±1.926
**Unassigned – No Feature[3]**	0.233 ±0.010	0.316 ±0.032	0.359 ±0.059	0.307 ±0.050	0.173 ±0.036	0.111 ±0.016	0.107 ±0.013
**Unassigned - Ambiguous[4]**	0.607 ±0.161	0.792 ±0.076	0.655 ±0.058	0.562 ±0.080	0.415 ±0.062	0.224 ±0.037	0.250 ±0.050
**After rRNA Removal**	2.897 ±0.234	4.423 ±0.118	4.265 ±0.443	3.745 ±0.246	3.151 ±0.068	2.729 ±0.369	2.787 ±0.237
**Features**							
CDS	2.617 ±0.231	4.107 ±0.087	3.978 ±0.415	3.456 ±0.278	2.858 ±0.050	2.425 ±0.305	2.487 ±0.257
tRNA	0.004 ±0.001	0.006 ±0.000	0.005 ±0.001	0.005 ±0.001	0.003 ±0.000	0.003 ±0.000	0.001 ±0.000
tmRNA	0.195 ±0.008	0.223 ±0.024	0.216 ±0.024	0.217 ±0.017	0.222 ±0.015	0.236 ±0.048	0.237 ±0.034
ncRNA	0.082 ±0.003	0.086 ±0.011	0.066 ±0.008	0.067 ±0.002	0.067 ±0.009	0.066 ±0.017	0.062 ±0.004

[1]Counts shown are the average of three biological replicates ± SEM
[2]Reads where only one end of the read pair mapped are removed from further analyses and are included in the unmapped read count
[3]Reads that mapped to regions that do not overlap a feature (i.e., intergenic regions)
[4]Reads that mapped to regions overlapping two or more features

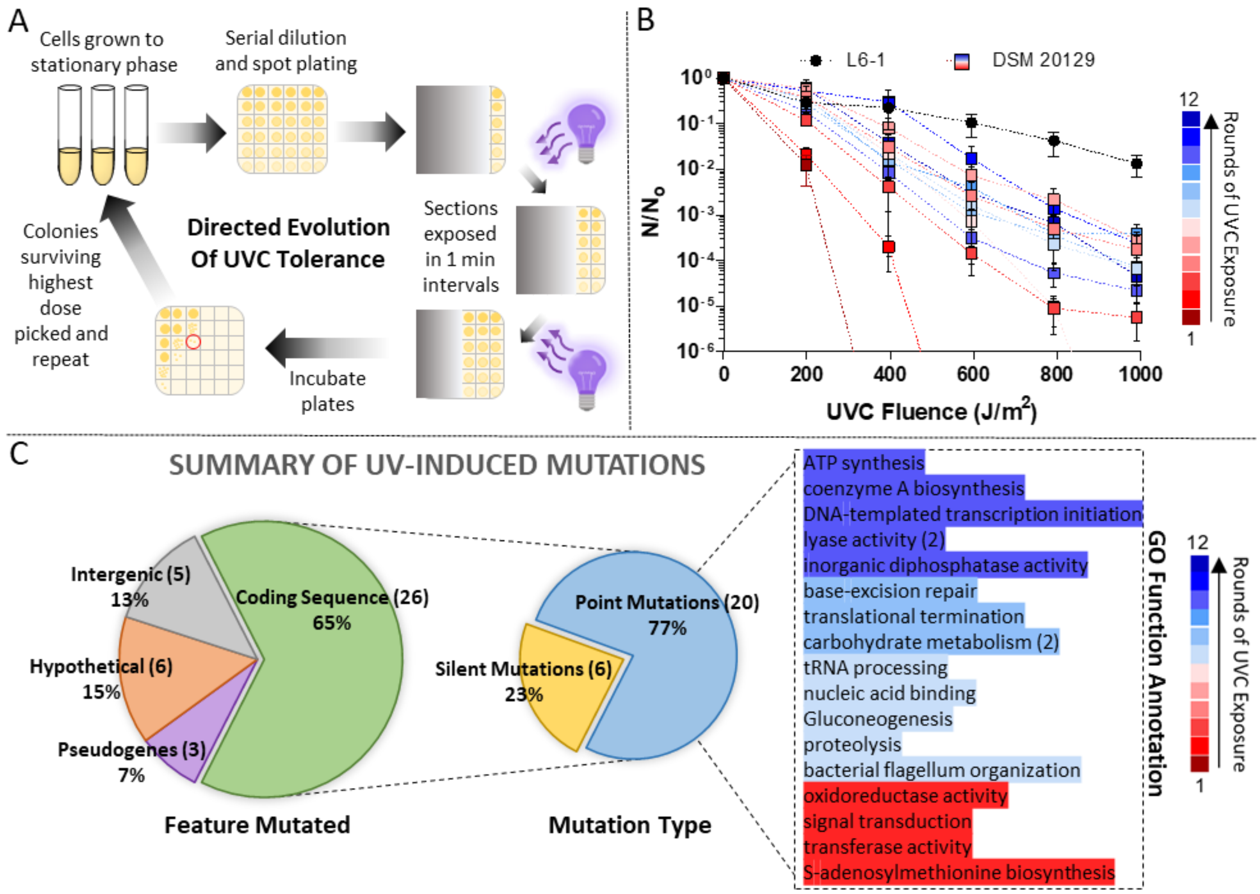

Figure S1: Directed evolution of UVCR tolerance in strain DSM 20129. A) Schematic depiction of the directed evolution method used to improve UVCR tolerance in strain DSM 20129. The details of the procedure are descried in the text. **B)** UVCR tolerance of DSM 20129 after each round of UVCR exposure as compared to isolate L6-1, measured as the number of surviving CFUs (N) at each UVCR dose divided by total number of CFUs in the unexposed control (N_0). Data shown are the average of three independent replicates. Error bars represent SEM. **C)** Summary of the mutations acquired during the directed evolution process. Mutation type (smaller pie chart) refers only to the mutations occurring in coding sequences (green slice in larger pie chart). Gene Ontology (GO) function annotations were determined for proteins that acquired amino acid point mutations in their coding sequence (blue slice in smaller pie chart).

A)

```
EcPhrB          MT----THLVWFRQDLRLHDNLALAAACRNSSARVLALYIATPRQWATHNMSPRQAELIN
AfPhrA          MSLKTAPVIVWFRKDLRLSDNLALLAAVEHGGP-VIPVYI-REKSAG--PLGGAQEWWLH
L61phr2         MS----GSVVWLRDDLRIADNPALRSAVDRGGP-VTVVYVLDEQSDGIRPFGAAARWWLH
DSM20129phr     MT----GAIVWLRDDLRLSDNPALRAAIDHGGD-VTVVVVLDEQSPGLRPIGAASRWWLH
                *:      :**:*.***: ** ** :*  ...  *  : :   :. .   :.      ::

EcPhrB          AQLNGLQIALAEKGIPLLFREVDDFVASVEIVKQVCAENSVTHLFYNYQYEVNERARDVE
AfPhrA          HSLAALSSSLEKAGGRLVLASGD----AERILRDLISETGADTVVWNRRYDPTGMATDKA
L61phr2         QSLAALDTDLRAHGSALVLRRGP----AAEVIPSLVQESGADAVFWNRRYGKVEREIDTG
DSM20129phr     HSLTALDADLRERGSRLVLRRGA----AATAIDQLVADTGADAVYWNRRYGAVERDIDAG
                 .* .*.  *   *  *::        :   : .:  :...  : :* :*       *

EcPhrB          VERALRN--VVCEGFDDSVILPPGAVMTGNHEMYKVFTPFKNAWLKRLREGM--------
AfPhrA          LKQKLRDDGLTVRSFSGQLLHEPSRLQTKSGGPYRVYTPF---W--RALEGSDEPHAPAD
L61phr2         IKSGLVESGIDAHSAAATLLWEPWTVLTGKGEPFKVFTPF---W--RAAQAMPEPRHPVP
DSM20129phr     IKAALHDRGVEAHSFAANLLWEPWTVLTGQGEPYKVFTPF---W--NAVQALPEPRHPLP
                ::  * :  :  ..    ::  *  : * .    ::*:***   *  .  :.

EcPhrB          -PECVAAPKVRSSGSIEPSPSITLNYPRQSFDTAH-FPVEEKAAIAQLRQFCQNGAGEYE
AfPhrA          PPKSLTAPKVWPKSEKLSNWKLLPTKPDWAKDFSDIWTPGETGALDKLDDFIDGALKGYE
L61phr2         EPETIAAPAD-VHSDALDDWALLPTTPDWAGGMRDAWTPGEHGAWDRMERFIDEALADYD
DSM20129phr     DPDDLPAPAD-VTGDALADWALLPTKPDWAGGLRDAWTPGEHGAQQQLEHFVEHALEDYD
                 *. :.**      ..   .  :  . *  : .  . :.  * .*  ::  * : .   *:

EcPhrB          QQRDFPAVEGTSRLSASLATGGLSPRQCLHRLLAEQPQALDGGAGSVWLNELIWREFYRH
AfPhrA          EGRDFPAKPATSLLSPHLAAGEISPAAVWHA-TKGLSRHIASNDISRFRKEIVWREFCYH
L61phr2         Q-RDEPAQAATSSLSPHLRWGEISPYQVWHR-LHETLEPDQRRAAPAFLRQLAWREFNWN
DSM20129phr     Q-RDEPAMEATSSLSPFLRWGEISPYQVWHR-LHERLEPAQRQNAAAFLRQLAWREFNWN
                : ** **  .** **. *  * :**    *         .       . : .:: ****  :

EcPhrB          LITYHPSLCK---HRPFIAWTDRVQWQS-NPAHLQAWQEGKTGYPIVDAAMRQLNSTGWM
AfPhrA          LLFHFPELGEKNWNDSF----DAFSWRD-DEKSFKAWTRGMTGYPIVDAGMRQLWQHGTM
L61phr2         EYFHCDDIARTNVRGEF----DAFPWRDASDDELDRWRAGTTGFDLVDAGMRELWHTGAM
DSM20129phr     EYFHCDDIATVNVRRNF----DAFPWRDPEAAELDRWRQGLTGFDLVDAGMRELWHSGAM
                   :  .:     .  *     * . *:. .    :. *  * **: :***.**:*   * *

EcPhrB          HNRLRMITASFLVKDLLIDWREGERYFMSQLIDGDLAANNGGWQWAASTGTDAAPYFRIF
AfPhrA          HNRVRMIVASFLIKHLLIDWRKGEKWFRDTLVDADPASNAANWQWVAGSGADASPFFRIF
L61phr2         HNRVRLATASFLVKNMLVDWRIGEQWFWDTLVDADAANNAANWQWVAGSGFDAAPYFRVF
DSM20129phr     HNRVRLAAGSFLVKNLLVDWRVGEQWFWDTLVDADPANNAANWQWVAGSGFDAAPYFRVF
                ***:*: ..***:*.:*:*** **::* . *:*.* * * ..***.*.:* **:*:**:*

EcPhrB          NPTTQGEKFDHEGEFIRQWLPELRDVPGKVVHEPWKWA----QKAGVTL--DYPQPIVEH
AfPhrA          NPILQGEKFDGDGDYVRRFVPELEKLERKYIHKPFEAPKDALKKAGVELGKTYPLPIVDH
L61phr2         NPDRQLERFDPHREYVRRWVPADE---------------------------DRPEPMLDL
DSM20129phr     NPDRQLERFDPHREYVHRWVPADE---------------------------LRPEPMVDL
                **  * *:** . ::::::*  .                            * *:::

EcPhrB          KEARVQTLAAYEAARKGK
AfPhrA          GKARERALAAYAAVKKTT
L61phr2         KATRQRALDAYADMRRA-
DSM20129phr     KASRQRALDAYERMRRS-
                  :* ::* **   ::
```

X = Nucleotide/FAD binding X = Electron transfer to DNA lesion

Figure S2: Amino acid sequence alignments for the photolyases found in strains L6-1 and DSM 20129 with known bacterial **A)** CPD photolyases and **B)** 6-4 photolyases.

B)

```
AfPhrB          M--SQLVLILGDQLSPSIAALDGVDKKQD-TIVLCEVMAEASYVGHHKKKIAFIFSAMRH
CsCryB          M--TRLILVLGDQLSDDLPALRAADPAAD-LVVMAEVMEEGTYVPHHPQKIALILAAMRK
Vc(64)FeS-BCP   MRYSVVRLILGDQLNHAHSWFS--EHRDDVLYLIAELHQEQEYVRHHIQKQCAFFAAMQA
L61Phr1         ------------------------------MVVVEAREFFTARPVHRTKAHLWLSALRH
                                              :: *           *  *    ::*::

AfPhrB          FAEELRGEGYRVRYTRIDDADNAGSFTGEVKRAIDDLTPSRICVTEPGEWRVRSEMDGFA
CsCryB          FARRLQERGFRVAYSRLDDPDTGPSIGAELLRRAAETGAREAVATRPGDWRLIEALE---
Vc(64)FeS-BCP   FADYLSAEGHHVWHLDLDASAQYNDLPDLIAQICQQVQADAFQYQRPDEYRLLEQMANLR
L61Phr1         RVRALGDRAEHVRVDRLRDA---------------LVGQHDLEVVDPPSRVLRGQVR---
                 .  *  .. :*    :  .                           * .  :    :

AfPhrB          GAFGIQVDIRSDRRFLSSHGEFRNWAAGR-KSLTMEYFYREMRRKTGLLMNGE-QPVGGR
CsCryB          -AMPLPVRFLPDDRFLCPADEFARWTEGR-KQLRMEWFYREMRRRTGLLMEGD-EPAGGK
Vc(64)FeS-BCP   -LSGITIGCVDTEHFLLPFAEIPEQFPAS-KAVLMEHFYRRMRKRFGYLMTADGKPEGGQ
L61Phr1         -HWGHDTTVLPSRGFVTSEDDFAAWAAGRGGRFLMETHYERVRRREGWLMEHD-APVGGA
                              *: .  ::      .    . ** .*..:*:: * **  :  * **

AfPhrB          WNFDAENRQP--ARPDLLR-PK---HPVFAPDKITKEVIDTVERLFPDNF-GKLENFGFA
CsCryB          WNFDTENRKP--AAPDLLR-PR---PLRFEPDAEVRAVLDLVEARFPRHF-GRLRPFHWA
Vc(64)FeS-BCP   WNFDADNRNK-LKSPDLLQLPT---PLCF--DNPVASIKARIERHRIPSIGQVGESLLWP
L61Phr1         FSLDAQNRQPPPRGATTLGLPDPWWPTEDEVDDEVRADLDRWERDGLVHFVGRDDRRRFA
                :.:*::**:      .  *  *            *  .       *       :      :.

AfPhrB          VTRTDAERALSAFIDDFLCNFGATQDAMLQDDP---NLNHSLLSFYINCGLLDALDVCKA
CsCryB          TDRAEALRALDHFIRESLPRFGDEQDAMLADDP---FLSHALLSSSMNLGLLGPMEVCRR
Vc(64)FeS-BCP   INRAQALSLLAHFCQICLPNFGRFQDAMTAQHPHRWSLYHSRLSFALNSKLLSPREVIEA
L61Phr1         VTPDEARAALEDFVAVRLGDFGPYEDAVLTND---WTMAHSLLSVPLNLGVLDPRDAVGA
                    :*   *  *    *  **  **:  :.     : *: **  :*  :*.. :.

AfPhrB          AERAYH--EGGAPLNAVEGFIRQIIGWREYMRGIYWLAGPDYVDS-NFFENDRSLP----
CsCryB          AETEWR--EGRAPLNAVEGFIRQILGWREYVRGIWTLSGPDYIRS-NGLGHSAALP----
Vc(64)FeS-BCP   TISAYRAAQGQISLAQVEGFVRQILGWREYVRGMYWSNMPHYQTR-NHLGAQRPLP----
L61Phr1         AVAAYE--SGAAPLASVEGFVRQIAGWRDYVWHLYWWLGDEYGADVEALGARHPGPEAHD
                :   :.  .*  .*  ****:*** ***:*:  ::     .*      : :     . *

AfPhrB          -----------VFYW----TGKTHMNCMAKVITETIENAYAHHIQRLMITGNFALLAGID
CsCryB          -----------PLYW----GKPTRMACLSAAVAQTRDLAYAHHIQRLMVTGNFALLAGVD
Vc(64)FeS-BCP   -----------SYFW----NGQTKMRCLQQAITQSLDFGYAHHIQRLMVTGNFALLTECD
L61Phr1         GSRSAGGGTGLPAWWRTLDAGDVEAVCLSVALDGVRDHGWLHHIQRLMVLGNWALQRGYD
                             :*        ..  *:  .:    : .: *******: **:**     *

AfPhrB          PKAVHRWYLEVYADAYEWVELPNVIGMSQFADGGFLGTKPYAASGNYINRMSDYCDTCRY
CsCryB          PAEVHEWYLSVYIDALEWVEAPNTIGMSQFADHGLLGSKPYVSSGAYIDRMSDYCRGCAY
Vc(64)FeS-BCP   PDQVDAWYLGIYIDAIEWVELPNTRGMALFADGGLIATKPYSASGSYINKMSDYCASCAY
L61Phr1         PVQLTEWFTDVFVDGTDWVMPANIIGMSQHADGGVVATKPYAAGGRYIDRMTDHCGGCRF
                *  :  *:  :: *. :**  .*  **: .** *:.:***  :.* **::*:*:*  * :

AfPhrB          DPKERLGDNACPFNALYWDFLARNREKLKSNHRLAQPYATWARMSEDVRHDLRAKAAAFL
CsCryB          AVKDRTGPRACPFNLLYWHFLNRHRARFERNPRMVQMYRTWDRMEETHRARVLTEAEAFL
Vc(64)FeS-BCP   QVKLKSGEKACPLNSLYWRFMLKHRDRLANNPRIGMLYKTWDKMTSDSQQAILSTADAYL
L61Phr1         DPTKRLGPDACPFTAGYWAFLERAEPALRRNPRMHRPLQQMRAM-PDLEQVVAQEAARRM
                  . : *  ***:.  ** *: : .  :  * *:          *    .  :   *   :

AfPhrB          RKLD-----
CsCryB          GRLHAGEPV
Vc(64)FeS-BCP   SQIES---L
L61Phr1         P--------
```

X = 4Fe-4S binding X = Nucleotide/FAD binding X = Chromophore binding

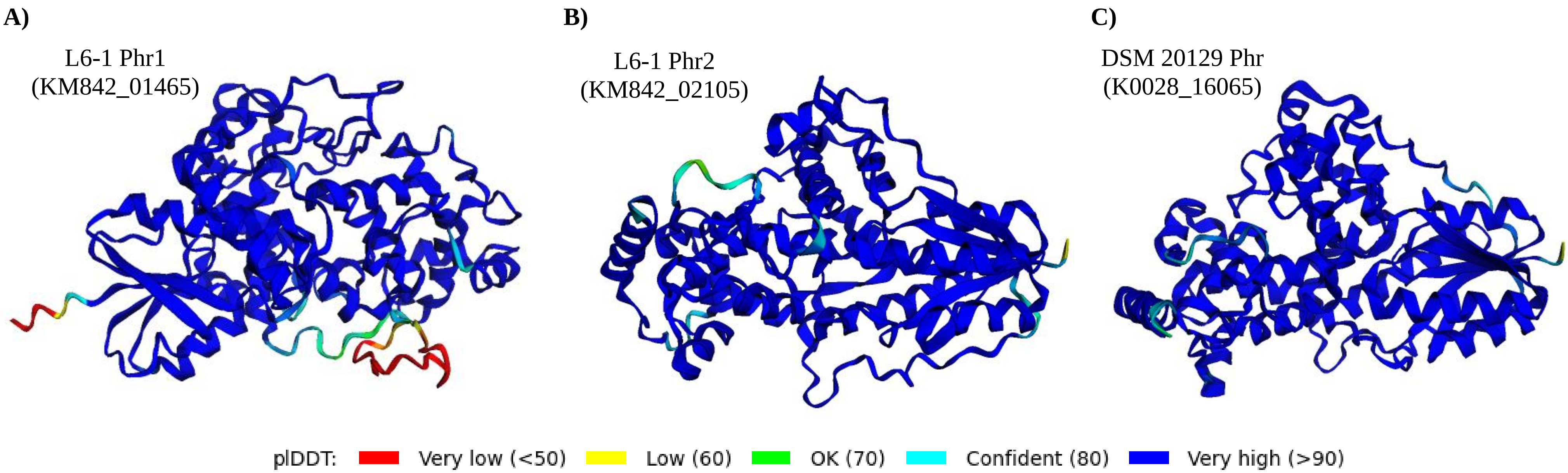

Figure S3: AlphaFold2 modeled structures for **A)** L6-1 Phr1 (KM842_01465) **B)** L6-1 Phr2 (KM842_02105) and **C)** DSM 20129 Phr (K0028_16065). Colors represent modeling confidence according to predicted local distance difference test (pLDDT) score.

A)

Alignment	Equivalent Residues	Sequence Identity	RMSD	TM-Score
L6-1_Phr2 vs. EcPhrB (1DNP)	438	32%	2.63	0.87
DSM_Phr vs. EcPhrB (1DNP)	438	34%	2.59	0.87

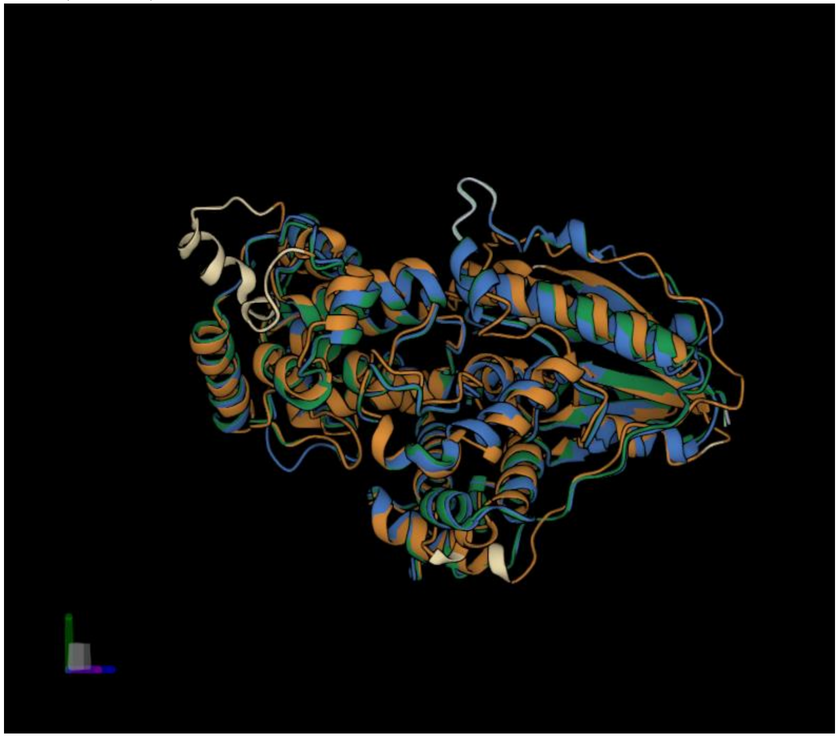

B)

Alignment	Equivalent Residues	Sequence Identity	RMSD	TM-Score
L6-1_Phr1 vs. AfPhrB (5KCM)	476	36%	1.84	0.89

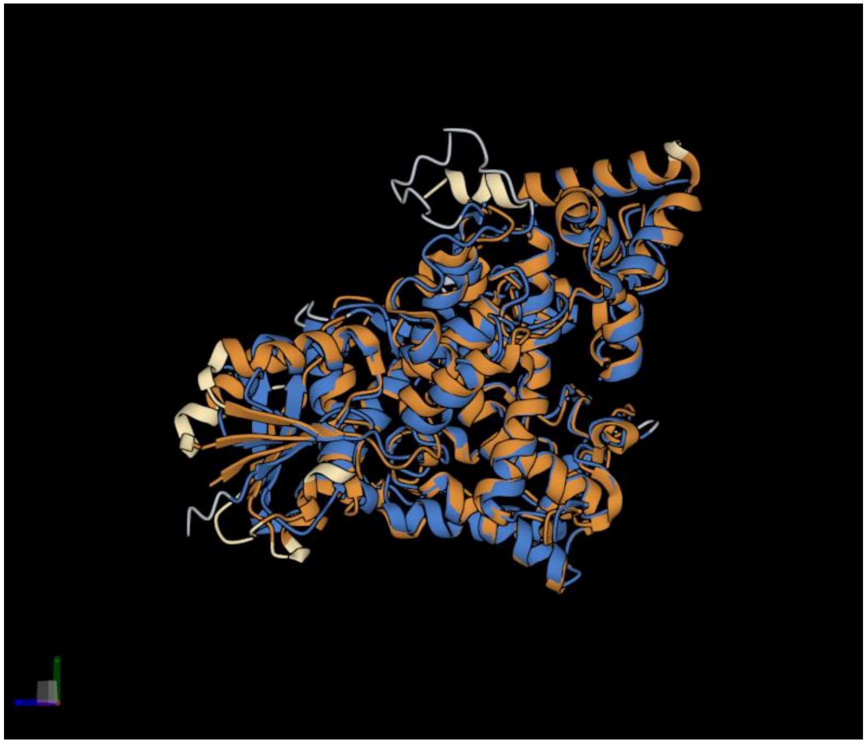

Figure S4: Structural alignments for the photolyases found in strains L6-1 and DSM 20129 with known bacterial CPD photolyases and 6-4 photolyases. A) Structural alignment and scores of CPD photolyase from *E. coli* (PDB ID: 1DNP, orange) with the predicted structures for photolyases in strains L6-1 (*phr2*: KM842_02105, blue) and DSM 20129 (*phr*: K0028_16065, green). **B)** Structural alignment of (6-4) photolyase from *A. fabrum* (PDB ID: 5KCM, orange) with the predicted structure for additional photolyase in strain L6-1 (*phr1*: KM842_01465, blue).

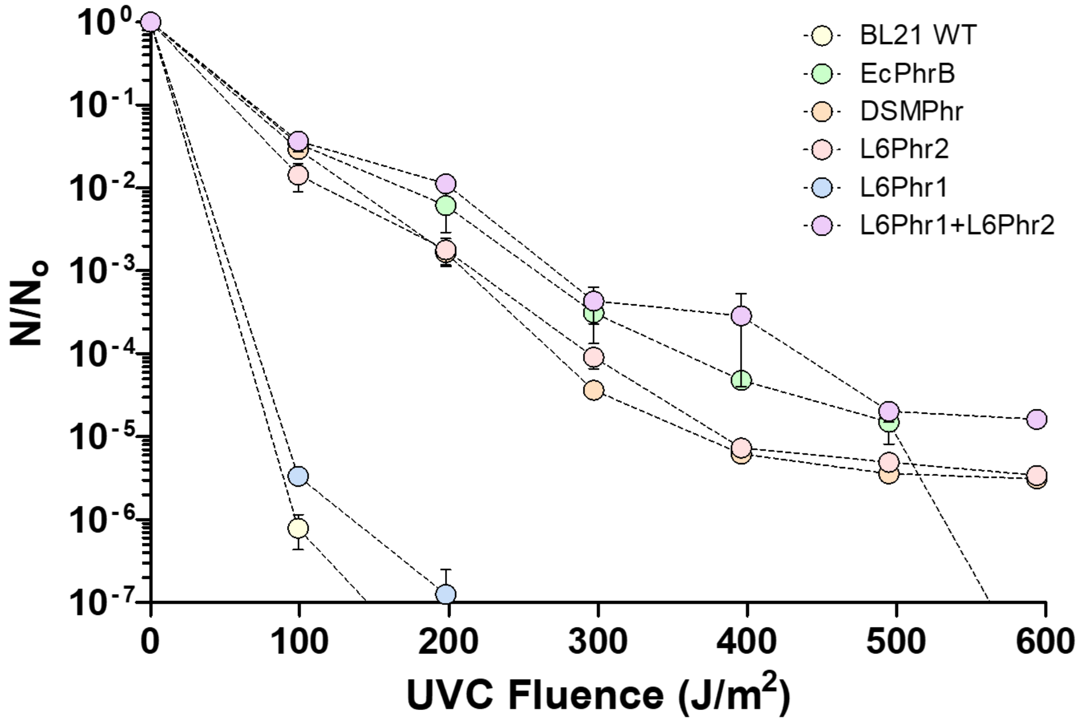

Figure S5: Photolyase activity determination by heterologous expression in *E. coli* BL21. UVCR survival of BL21 cells expressing either the L6-1 *phr2* (L6Phr2), L6-1 *phr1* (L6Phr1), both L6-1 photolyase genes (L6Phr1 + L6Phr2), or the DSM 20129 *phr* (DSMPhr) under photoreactivating (light) conditions. Expression of the *E. coli* CPD photolyase from wild type BL21 cells (BL21 WT) and overexpression from the pDLx expression vector (EcPhrB) were used as controls. The average of three independent replicates with error bars representing SEM are plotted.

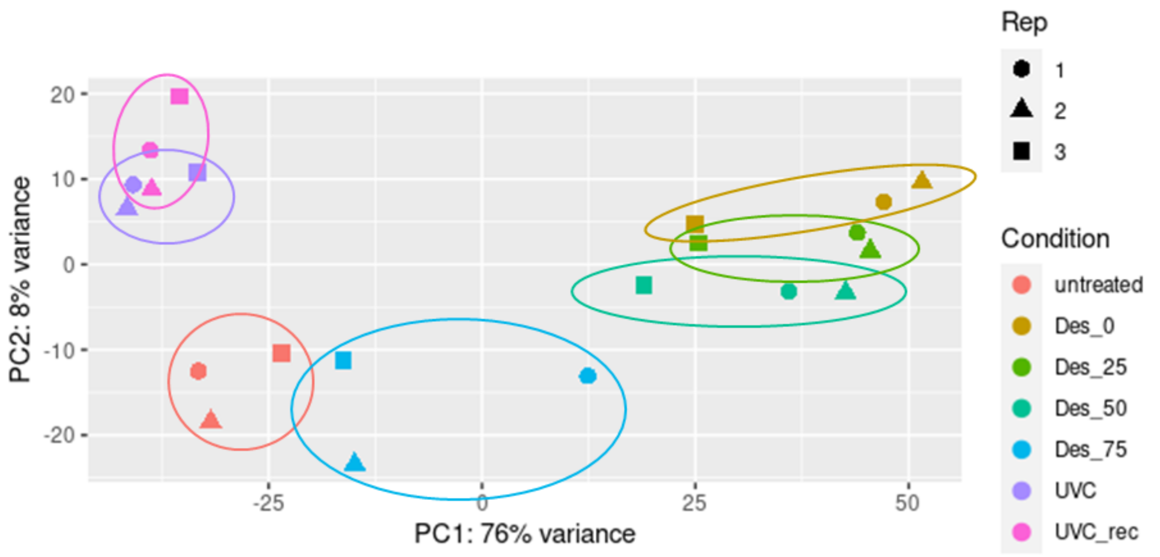

Figure S6: Principal component analysis of RNA-seq data. Results shown as a two-dimensional scatter plot of the first two principal components of the data. Each point represents a sequencing sample and samples with similar gene expression profiles are clustered together. Biological replicate number is indicated by the shape of each point and treatment condition is indicated by color (untreated = time zero control; Des_75, Des_50, Des_25, and Des_0 = cells dehydrated to 75%, 50%, 25%, and 0% of initial water content, respectively; UVC = UVCR treated cells with no recovery time, UVC_rec = UVCR treated cells with a 20 min recovery). Ellipses illustrate the clustering of replicate samples.
